# Mindfulness-based crisis interventions (MBCI) for psychosis within acute inpatient psychiatric settings; a feasibility randomised controlled trial

**DOI:** 10.1186/s12888-020-02608-x

**Published:** 2020-04-29

**Authors:** Pamela Jacobsen, Emmanuelle Peters, Emily J. Robinson, Paul Chadwick

**Affiliations:** 1 Department of Psychology, King’s College London, Institute of Psychiatry, Psychology and Neuroscience (IoPPN), De Crespigny Park, London, SE5 8AF UK; 2 Department of Psychology, University of Bath, Bath, BA2 7AY UK; 3 South London and Maudsley NHS Foundation Trust, Bethlem Royal Hospital, Monks Orchard Road, Beckenham, Kent BR3 3BX UK; 4 Department of Biostatistics & Health Informatics, King’s College London, Institute of Psychiatry, Psychology and Neuroscience (IoPPN), De Crespigny Park, London, SE5 8AF UK; 5 Present address: School of Population Health and Environmental Sciences, King’s College London, London, SE1 1UL UK

**Keywords:** Psychiatric units, Psychosis, Psychotherapy, Mindfulness, Crisis intervention, Psychiatric hospital readmission

## Abstract

**Background:**

Inpatient psychiatric care is a scarce and expensive resource in the National Health Service (NHS), with chronic bed shortages being partly driven by high re-admission rates. Brief inpatient talking therapies for psychosis could help reduce re-admission rates. The primary aim was to assess feasibility and acceptability of a novel, brief, mindfulness-based intervention for inpatients with psychosis. The secondary aim was to collect pilot outcome data on readmission rate, at 6 and 12 months (m) post discharge, and self-report symptom measures at 6 m.

**Methods:**

The amBITION study (BrIef Talking therapIes ON wards) was a parallel group, feasibility randomised controlled trial (RCT). In addition to treatment as usual (TAU), eligible inpatients with psychotic symptoms were randomly allocated to receive either (Mindfulness-Based Crisis Intervention; MBCI) or a control intervention (Social Activity Therapy; SAT), for 1–5 sessions.

**Results:**

Fifty participants were recruited (26 MBCI; 24 SAT); all received at least 1 therapy session (mean = 3). Follow-up rates were 98% at 6 m and 96% at 12 m for service use data extracted from clinical notes, and 86% for self-report measures. At 6 m follow-up, re-admission rates were similar across groups (MBCI = 6, SAT = 5; odds ratio = 1.20, 95% CI: 0.312–4.61). At 12 m follow-up, re-admissions were lower in the MBCI group (MBCI = 7, SAT = 11; odds ratio = 0.46, 95% CI: 0.14–1.51). Three participants experienced adverse events; none was related to trial participation.

**Conclusions:**

Delivering a brief mindfulness-based inpatient intervention for psychosis is feasible and acceptable, and may reduce risk of short-term readmission. These promising findings warrant progression to a larger clinical effectiveness trial.

**Trial registration:**

ISRCTN37625384.

## Background

Inpatient psychiatric care is a costly and scarce resource in global healthcare [1], and demand for beds often outstrips supply in the United Kingdom (UK) [2]. Given the scarcity of inpatient beds, the clinical threshold for psychiatric admission is very high, and most people admitted to hospital in National Health Service (NHS) settings are experiencing psychotic symptoms [3]. The short-term re-admission rate is around 15% at 90 days post-discharge [4, 5]. Reducing short-term re-admission rates is therefore considered a key quality benchmark indicator for mental health care internationally [6]. This is because of the high economic burden of inpatient care, and the disruption re-admissions cause to a service user’s recovery after a mental health crisis. Psychological therapies may reduce the risk of short-term re-admission [7]; however, these are not widely offered to service users with psychosis during an inpatient admission [8], and there is considerable heterogeneity in type and delivery of therapy offered [9]. This is despite the fact service users report high levels of dissatisfaction with inpatient care [10, 11], and say they would like better access to psychological therapies during their admission [12]. Clinical guidelines from the UK’s National Institute for Health and Care Excellence (NICE) suggest that Cognitive-Behavioural Therapy for Psychosis (CBTp) may be started in the acute phase during an inpatient setting [13]. However, a standard course of 16 weekly sessions of CBTp is not a good fit for the typical treatment window of an inpatient admission, given that the average length of an acute inpatient admission in the UK is approximately 30 days [3]. There is therefore a clear need for briefer inpatient interventions, which are adapted for people experiencing an acute mental health crisis.

There have been two promising pilot trials in the United States (US) of an acceptance-based cognitive therapy (Acceptance and Commitment Therapy; ACT), specifically adapted to be delivered within inpatient settings [14, 15]. The adapted therapy is brief (1–5 sessions), with stand-alone sessions to accommodate unpredictable lengths of stay, designed to target the putative underlying psychological processes implicated in crisis such as experiential avoidance [16]. Pilot results indicated that the ACT intervention reduced the risk of re-admission by approximately 50% at 4-month follow-up, compared to treatment as usual. Third-wave cognitive therapies including ACT, and mindfulness for psychosis [17], may be particularly suitable as crisis interventions, as they can help a person identify and modify maladaptive coping strategies during a key window for intervention. The present study is the first randomised controlled trial (RCT) to assess a mindfulness-based intervention for psychosis within an NHS acute inpatient setting. The objectives were to assess: (i) feasibility of implementing a brief mindfulness-based intervention within inpatient settings, (ii) whether patients and ward staff find it an acceptable intervention, (iii) to collect electronic health records data on readmission and relapse rates at 6-month and 12-month follow-up, and (iv) to collect self-reported clinical measures of symptoms and recovery at 6 months.

## Method

### Study design

This study was a single-centre, parallel-group, feasibility RCT. Service use and self-report measures were taken at baseline, end of therapy, 3- and 6-month follow-up (90 and 180 days post-discharge date respectively). The study was registered prospectively on the IRSCTN registry (ISRCTN37625384; BrIef Talking therapies ON wards (amBITION study). For full trial protocol see Jacobsen et al. [18]. As an amendment to the original protocol, the follow-up period for re-admission/relapse rate was extended up to 12-month follow-up, following the same procedures for data extraction and rating as at 6 month follow-up.

### Participants – inclusion/exclusion criteria

#### Inclusion criteria

i) Aged 18 or above.
ii) Current psychiatric inpatient on a working-age adult ward, with initial admission < 14 days ago.
iii) Diagnosis of schizophrenia-spectrum disorder or psychotic symptoms in the context of an affective disorder (ICD-10 codes F20–39).
iv) Reports at least one current positive psychotic symptom (scores > 1 on frequency on self-report symptom scale).
v) Able to give informed consent to participate in trial, as assessed by consultant psychiatrist/responsible clinician and researcher.
vi) Willing and able to engage in psychological therapy.

#### Exclusion criteria

i) established diagnosis of learning disability, or major cognitive impairment arising from any underlying medical condition (e.g. head injury, neurological disorder) resulting in significant functional impairment.
ii) unable to engage in a talking therapy in English, or to complete simple written questionnaires in English.
iii) primary diagnosis of substance misuse.
iv) lacks capacity to consent to participation in research trial.
v) unable to take part in individual therapy due to risk of aggression/violence.
vi) mental state precludes possibility of engaging in a talking therapy, e.g. significant thought disorder, as assessed by clinical team and researcher.

### Recruitment, randomisation and blinding

All consecutive new admissions from 4 acute inpatient wards (3 male, 1 female) in a psychiatric hospital in South London were screened for eligibility. All consecutive new admissions were screened for initial eligibility, defined as admittance to hospital within the last 14 days presenting with positive psychotic symptoms in the context of a psychosis or mood disorder. Potentially eligible patients were then approached to take part with permission of their inpatient Consultant Psychiatrist and the nursing team. Patients could take part in the trial if they were admitted under a section of the Mental Health Act (MHA) so long as they were deemed to have retained capacity to consent to participation in research. Further eligibility screening by reference to electronic clinical notes was conducted with written consent from patients who had been approached and were potentially interested in participating. All reasons for ineligibility, or reasons for declining to participate in the study, were recorded on the screening log. Patients were approached to participate by PJ, or a representative from the local Clinical Research Network (CRN). After giving written informed consent, eligible participants completed baseline measures. Each participant was then randomised at the beginning of their first therapy session, to minimise any delay between entry into the study and the start of the intervention. The randomisation sequence was generated by the Kings Clinical Trials Unit (KCTU), and randomisation was via an online computerised system. Block randomisation was used, with randomly varying block sizes to ensure allocation concealment. The randomisation sequence was not stratified.

As with all psychological therapy trials, both the therapist and participant were aware of the treatment condition they were randomised to. The participant’s inpatient and community care team were however blinded to treatment allocation. The service use data, which included relapse and re-admission assessed at 6-month follow-up, were blind rated by an appropriately trained researcher who was not otherwise involved in the trial. All self-report questionnaires were collected by PJ.

### Description and delivery of therapies

Therapy sessions in both conditions were delivered on an individual basis in a private room on the inpatient wards. PJ was the trial therapist in both conditions. PJ is a Clinical Psychologist registered with the UK Health & Care Professions Council (HCPC) with expertise in cognitive behavioural therapy for psychosis (CBTp) and mindfulness interventions as well as extensive experience of working in acute settings. Therapy sessions in both conditions ranged from 1 to 5 sessions, depending on variables such as length of admission, with the frequency of sessions adjusted as needed between a minimum of weekly and maximum of daily. All sessions followed a stand-alone, self-contained format, to accommodate unpredictable lengths of stay and unexpected discharges. A minimum ‘dose’ of therapy was defined as 1 therapy session. The treatment phase was restricted to the duration of the inpatient admission. All participants in the trial continued to receive treatment as usual (TAU) both during their inpatient admission and post-discharge, which could include medication, occupational therapy and any other psychosocial interventions offered as part of routine care.

#### Mindfulness-based crisis interventions (MBCI)-experimental intervention

MBCI was developed in line with the ACT trials conducted in the US and the model of mindfulness for psychosis proposed by Chadwick [19]. The treatment protocol for the current trial was adapted for use within an acute crisis setting, following Bach and Hayes [14] and Gaudiano and Herbert [15]. In brief, each session included 3 key components, with varying amounts of emphasis placed on each component depending on the session number and the stage of therapy. These were i) developing mindfulness skills (guided practice), ii) making sense of crisis using mindfulness model, iii) identifying values and setting goals.

The guided practice was always done at the beginning of each session. The first session focused primarily on the development of a crisis-focused formulation, using a standard template, which formed the basis of a shared understanding of what brought the person into hospital on this occasion. This formulation then informed any future sessions, focusing on key processes that had been identified in the run-up to the crisis, such as experiential avoidance. The therapist also worked with the participant to identify their values (e.g. family, work, health, society), and discuss specific behavioural goals consistent with these values. Participants were then helped to set a small, achievable goal for homework at the end of each session that could be reviewed at the beginning of next session, where possible. In preparation for discharge, longer-term goals were also identified (e.g. starting a college course) and were shared with the community care team at the end of therapy in the end of therapy letter, to act as a bridge to carrying on the recovery process in the community.

#### Social activity therapy (SAT) – control intervention

The control condition was taken from the PICASSO trial of CBTp for people with psychosis and a history of violence, which was conducted partly on inpatient wards [20]. SAT involved working collaboratively with the participant to identify activities they enjoyed and which they could engage in during and between sessions as they wished (e.g. board games, puzzles). The aim was to provide a supportive environment with a therapist using non-specific aspects of therapy (e.g. collaboration, feedback, empathy). The aim was to keep the sessions activity focussed, and to be supportive, collaborative and empathic without employing any techniques specific to any model of therapy, including CBTp or mindfulness-based therapies.

#### Treatment Fidelity

All participants were asked their permission to audio-tape sessions for the purposes of clinical supervision and fidelity checks. Fidelity checks were completed using the adherence and competency scale developed for the trial. The scale included 4 sub-scales; i) non-specific therapy factors, ii) MBCI-specific factors, iii) SAT specific factors, and iv) CBTp factors proscribed in both treatment conditions. Copies of the therapy manuals and adherence and competency scale are available from the authors on request.

### Outcome measures

#### Primary objective – feasibility/acceptability

1. Number of eligible participants identified over study period
2. Total numbers recruited into trial and recruitment rate (benchmark of 80% of target)
3. Proportion of participants who dropped out during the intervention stage
4. Range and average number of sessions completed (including number of sessions attended as a proportion of those offered)
5. Reasons for participants dropping out during the intervention stage
6. Number lost to follow-up and reasons (benchmark of less than 20% to be set in line with previous studies)
7. Any unexpected adverse effects of participating in the trial
8. Participant and staff feedback on trial procedures and therapy

#### Secondary objective - pilot data on clinical outcome measures

Service use outcomes were collected via electronic health records, where available, including for participants who did not complete questionnaire outcome data. The main outcome for service use data was re-hospitalisation at 6-month and 12-month follow-up (defined as ≥1 occupied bed day). Additional outcomes were time to re-admission, total number of occupied bed days in follow-up period, episodes of care with home treatment team, contact with community mental health team, reference to therapy goal, and relapse rate. Relapse was defined as an exacerbation in psychotic symptoms followed by a documented change in clinical management.

Measures of symptoms and recovery were collected via self-report questionnaires. A measure of therapy credibility was also taken at baseline, immediately after randomisation. Participants were read a brief description of the therapy they had been assigned to, and were then asked to rate on a scale from 0 (not helpful at all) to 10 (extremely helpful) how helpful they thought the therapy sounded. It is important to measure therapy credibility in psychological therapy trials, in order to rule out differential credibility as a reason for why outcomes may differ between treatment arms, as patients may be more likely to drop-out or not engage fully in therapies they rate as less credible. At the end of the trial, participants were also asked to rate their satisfaction with the therapy they had received, on a scale from 0 (not at all satisfied) to 10 (complete satisfied). Satisfaction was measured in order to assess whether this differed between treatment arms, and whether MBCI was rated as an acceptable intervention by participants overall. For full list and description of measures see Additional file 1 (Table 3).

### Sample size

The target recruitment was set at *N* = 60 (30 in each arm). This was determined with reference to existing studies in the field, and is consistent with good practice recommendations for feasibility studies, where sample sizes between 24 and 50 have been recommended [21, 22].

### Analysis plan

In line with the goals of the study, the analysis plan was focused on descriptive statistics for key feasibility outcomes. This included calculating proportion of target sample size achieved (≥80% benchmark), proportion of initially eligible patients who were randomised, and proportion of participants lost to follow-up (≤20% benchmark). For service use outcomes, the proportion of people readmitted to hospital in the follow-up period, with associated odds ratio and 95% confidence intervals (95% CI) was calculated, and survival curves presented using a Kaplan-Meier plot. For symptom and recovery questionnaire data (continuous data), descriptive statistics were first calculated based on unadjusted means. A general linear model, co-varying for baseline score and treatment condition, was then used to calculate group difference estimates and associated 95% CI. All analyses were conducted in accordance with intention to treat (ITT) principles, i.e. patients were analysed in the groups to which they were randomised.

### Procedure for recording and reporting adverse events

In addition to standard adverse events as specified by the NHS REC (research ethics committee) as requiring mandatory reporting (death, hospitalisation, disability, birth defect), several additional adverse events were identified in advance, and specified in the trial protocol, which were of particular relevance to this patient group and clinical setting. Taking this approach can be helpful in making adverse event reporting guidelines more relevant to psychological therapy trials as standard definitions of adverse events are focussed on occurrences of physical harms, designed primarily for drug trials. However, other events, such as emotional harms, or occurrences of potentially risky behaviour, are often more relevant to monitoring harm for psychological therapy trials. Additional adverse events were therefore identified in the trial protocol for this study, which consisted of self-harm, absconsion from the ward, and harm to or from others (e.g. assault). All adverse events were reported to the independent chair of the Trial Steering Committee, who ratified the project team’s assessment of whether they could be related to trial participation and would require reporting to the ethics committee and Trust R&D department.

## Results

### Primary objective – feasibility/acceptability

**Fig. 1:**
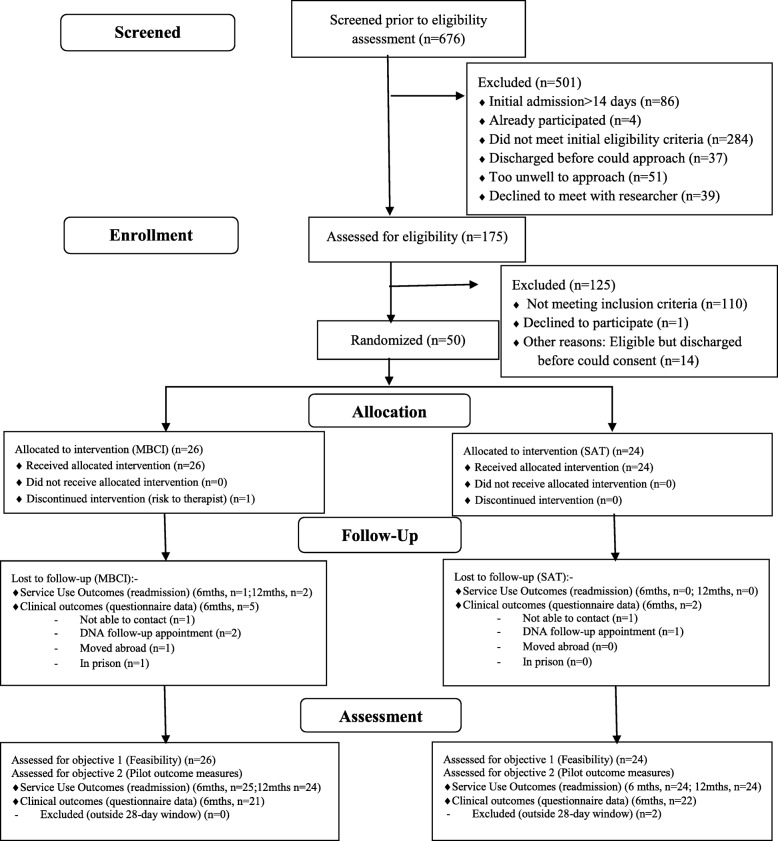
Flow through trial (CONSORT diagram)

Recruitment took place over 15 months from November 2015 to January 2017, with 12-month follow-ups completed by February 2018. Flow through the study is shown in Fig. 1. A total of 676 consecutive admissions were screened, of which 86 were classified as not ‘new admissions’ but inpatient transfers from other wards or hospitals, where the overall inpatient admission had started > 14 days ago. Of the remaining 590 acute admissions, *n* = 4 were re-admissions who had already participated in the trial, and *n* = 284 did not meet the initial eligibility criteria of presenting with positive psychotic symptoms in the context of a F20–39 diagnosis. This left 302/590 (51%) of new admissions who were screened further for eligibility. Of these, 37 were discharged before they could be approached, 51 were judged too unwell to approach by the clinical team, and 39 declined to meet with the researcher, leaving 175 patients who were assessed further. Of these 175, 65 were eligible to participate, with 14 being discharged before they could be randomised into the study, and 1 who declined to participate. This left fifty participants (77% of those eligible) who were randomised into the trial (83% of pre-set target).

Baseline demographic and clinical data for participants are shown in Table 1. No statistical significance tests or confidence intervals were calculated for the difference between randomised groups on any baseline variables, in line with the recommendations of Altman and Dore [23].

**Table 1:** Baseline demographic and clinical characteristics of participants

	SAT (*N* = 24)	MBCI (*N* = 26)	OVERALL (*N* = 50)
Age			
- Mean (range)	33 years (19–65)	35 years (18–52)	34 years (18–65)
Gender			
- Male	17 (71%)	17 (65%)	34 (68%)
- Female	7 (29%)	9 (35%)	16 (32%)
Ethnicity			
- White	8 (33%)	8 (30%)	16 (32%)
- Asian	3 (13%)	3 (12%)	6 (12%)
- Black	9 (37%)	12 (46%)	21 (42%)
- Mixed Race	3 (13%)	3 (12%)	6 (12%)
- Other	1 (4%)	0 (0%)	1 (2%)
Diagnosis			
- F20–29 (Schizophrenia-spectrum)	17 (71%)	20 (77%)	37 (74%)
- F30–39 (Mood disorder)	7 (29%)	6 (23%)	13 (26%)
Psychotic symptoms (self-report)			
- Delusions only	12 (50%)	14 (54%)	26 (52%)
- Voices only	1 (4%)	0 (0%)	1 (2%)
- Delusions + voices	11 (46%)	12 (46%)	23 (46%)
Legal status on admission			
- Informal	6 (25%)	8 (31%)	14 (28%)
- MHA^a^ Sec 2	13 (54%)	14 (54%)	27 (54%)
- MHA Sec 3	5 (21%)	3 (11%)	8 (16%)
- MHA Sec 37	0 (0%)	1 (4%)	1 (2%)
Open to secondary care psychiatric services on admission			
- Yes	12 (50%)	10 (38%)	22 (44%)
- No	12 (50%)	16 (62%)	28 (56%)
Years known to services			
- < 1 year	5 (21%)	4 (15%)	9 (18%)
- 1–5 years	4 (17%)	6 (23%)	10 (20%)
- 6–10 years	6 (25%)	8 (31%)	14 (28%)
- 11–15 years	2 (8%)	2 (8%)	4 (8%)
- > 15 years	7 (29%)	6 (23%)	13 (26%)
Psychiatric medication at baseline			
- Prescribed at least one medication	23 (96%)	26 (100%)	49 (98%)
- Prescribed an anti-psychotic	21 (88%)	25 (96%)	46 (92%)
- Prescribed an anti-depressant	4 (21%)	5 (19%)	9 (18%)
- Prescribed a mood-stabilizer	4 (21%)	1 (4%)	5 (10%)
Anti-psychotic medication dose at baseline			
- Mean % of BNF maximum dose	51.2%	49.6%	50.3%
- 95% CI	[37.7, 64.6]	[39.5, 59.7]	[42.4–58.2]
- Range	16.5–137.5	7.5–100	7.5–137.5
Previous admissions			
- Yes	14 (58%) (mean = 5.64, range 1–14)	21 (81%) (mean = 4.00, range 1–10)	35 (70%) (mean = 4.66, range 1–14)
- No	10 (42%)	5 (19%)	15 (30%)
Admission in previous 12 months			
- Yes	7 (29%)	8 (31%)	15 (30%)
- No	17 (71%)	18 (69%)	35 (70%)
Psychological therapy in past 5 years			
- None	11 (46%)	12 (46%)	23 (46%)
- Offered	3 (12%)	4 (15%)	7 (14%)
- Received	10 (42%)	10 (39%)	20 (40%)

^a^*MHA* Mental Health Act, *Sec 2* Section 2 (assessment), *Sec 3* Section 3(treatment), *Sec 37* Section 37 (hospital order)

#### Description and acceptability of treatment

All participants received at least one therapy session, and no-one dropped out during the intervention stage. The mean number of sessions completed was 3 (range 1–5) in both arms of the trial. Overall, 76% of offered appointments were attended (146/191 sessions). Therapy credibility, assessed just after randomisation on scale of 0–10, where 10 is extremely helpful, was high in both treatment conditions (MBCI = 7.77, SAT = 7.71), and did not differ significantly between treatment condition (t (48) = − 0.09, *p* = 0.93). Overall satisfaction with therapy received was high in both MBCI (mean = 9.11, SD = 1.1) and SAT groups (mean = 8.27, SD = 1.91) on a 0–10 scale, where 10 = completely satisfied. In terms of treatment as usual (TAU), virtually all participants (49/50; 98%) were prescribed at least one medication, most commonly an anti-psychotic. Rates of psychological intervention as part of inpatient TAU was very low. No participants, in either arm of the trial, received any additional individual psychology sessions, and only 6/50 (12%) attended a therapy group on the ward (MBCI 4; SAT 2). In the MBCI group, people reported that the most helpful things about the therapy was having someone supportive to talk to, and getting help to make sense of what was going on at a confusing time*.* Some people specifically referred to the mindfulness exercises in saying what they found most helpful:-

“*Liked the mindfulness component which I found specifically helpful*”

“*Mindfulness exercise – felt ‘trapped’ but felt I could get over it and not feel trapped. Taking time out – step out of the box and have a breather*.”

In addition, 8 staff members across all participating wards completed individual feedback interviews (3 consultant psychiatrists, 3 ward managers, & 2 staff nurses). Staff commented on the importance of patients having access to psychological therapies on the ward, whilst also acknowledging that routine access to such therapies was usually limited:-

“*I think anything which is an additional or optional treatment or getting people engaged in something psychological and therapeutic is a good thing […] They don’t have routine access to psychological therapies.* (Consultant Psychiatrist)

“*I’ve been in this environment for a few years now and what I found when I came into it was there isn’t enough of this kind of stuff, there’s a lack of it and I think a lot of that’s got to do with it being acute so talking therapies and stuff aren’t always – they’re more ad-hoc.”* (Staff Nurse)

#### Treatment fidelity

Consent to taping of at least one therapy session was high (36/50; 74%). Twenty sessions from the 108 total available sessions were randomly selected (10 from each condition), including sessions from both earlier and later stages in treatment, for fidelity ratings. The independent rater, who was blind to treatment condition, was a senior Clinical Psychologist, with many years’ experience of training and assessing competencies in CBT for psychosis and was not otherwise involved in the trial. All 20 sessions were correctly identified as coming from either a SAT or MBCI session. Fidelity to treatment model was 100% across all sessions rated, and competency was at least satisfactory for all therapy components that were present within a session.

#### Retention at follow-up

At 6-month follow-up, only one participant was lost to follow-up as they moved abroad immediately upon discharge. Data on hospital re-admission were available for the remaining 49 participants (98% follow-up). Follow-up rate for self-report questionnaire measures was 86%, which exceeded the 80% benchmark set in the trial protocol. An additional person was later lost to follow-up, after they were released from prison (96% follow-up at 12 months on re-admission data). See Fig. 1 (CONSORT diagram) for break-down of numbers lost to follow-up at each time-point).

#### Adverse events

Three participants (2 SAT; 1 MBCI) experienced a total of 5 adverse events over the course of the trial (these included 1 minor assault, 3 medication overdoses not requiring treatment, and 1 accidental fall). None was related to trial participation.

### Secondary objective - pilot data on clinical outcome measures

Service use outcomes at 6-month follow-up are summarised in Table 2. In the 6 months after admission, a total of 11 participants were re-admitted to hospital with approximately equal re-admission rates between groups (MBCI = 6, SAT = 5; odds ratio = 1.20, 95% CI: 0.312–4.61). Episodes of care with home treatment teams (HTTs) were relatively rare as stand-alone episodes of care (i.e. not overlapping with an inpatient admission). Only 2 participants in the trial had HTT involvement, but did not require inpatient admission (both in the SAT group). Relapse rates were also similar between the treatment groups (MBCI = 6, SAT = 7; odds ratio = 0.81, 95% CI: 0.26–2.90). In most cases there was no difference between readmission/relapse ratings, as most relapses of psychotic symptoms resulted in an inpatient admission.

**Table 2:** Service use outcomes at 6-month follow-up by treatment condition

Outcome	6-month follow-up		12-month follow-up	
	MBCI (***N*** = 25^**a**^)	SAT (***N*** = 24)	MBCI (***N*** = 24^**b**^)	SAT (***N*** = 24)
**1) Re-hospitalisation (≥1 OBD**^**c**^**)**				
- Yes	6 (24%)	5 (21%)	7 (29%)	11 (46%)
- No	19 (76%)	19 (79%)	17 (71%)	13 (54%)
**2) Mean survival time (days)**				
- Mean (SE)	156 (8.9)	164 (8.0)	291 (23.8)	278 (23.1)
- Range	41–180	58–180	41–360	58–360
- 95% CI	139–174	148–179	244–337	233–323
**3) Total number of OBDs**				
- Mean (SD)	11 (26)	14 (36)	28 (66)	33 (63)
- Range	0–83	0–117	0–244	0–212
- 95% CI	0–22	0–28	0–56	7–60
**4) Episodes of care with crisis/home treatment team (HTT)**^**d**^				
- Yes	0 (0%)	2 (8%)	2 (8%)	4 (17%)
- No	25 (100%)	22 (92%)	22 (92%)	20 (83%)
**5) No. of contacts with CMHT**^**e**^				
- Mean (SD)	14 (7)	13 (7)		
- Range	0–32	3–34		
- 95% CI	10–17	10–16		
**6) Reference to therapy goal, which was shared with team**				
- Yes	21(88%)			
- No	3 (12%)			
**7) Relapse**				
Exacerbation in psychotic symptoms + change in clinical management				
- Yes	6 (25%)	7 (29%)	9 (38%)	14 (58%)
- No	18 (75%)	17 (71%)	15 (62%)	10 (42%)

^a^Re-admission data not available for 1 participant

^b^Re-admission data not available for 2 participants

^c^*OBD* Occupied bed day

^d^Stand-alone episodes of care only (i.e. not overlapping with inpatient admissions)

^e^*CMHT* Community mental health team; no. of contacts excluding therapy appointments

Full descriptive statistics of self-report questionnaire measures of symptoms and recovery are presented in Additional file 1 (Table 4). In summary, psychotic symptoms and mood symptoms (depression, anxiety, and stress) in both groups showed the expected improvement over the course of the inpatient admission from baseline to end of therapy, with no indication of differential improvement between the groups. There was a flattening of scores over the 6-month follow-up period in both groups, indicating little additional improvement in the short-term after discharge.

**Fig. 2:**
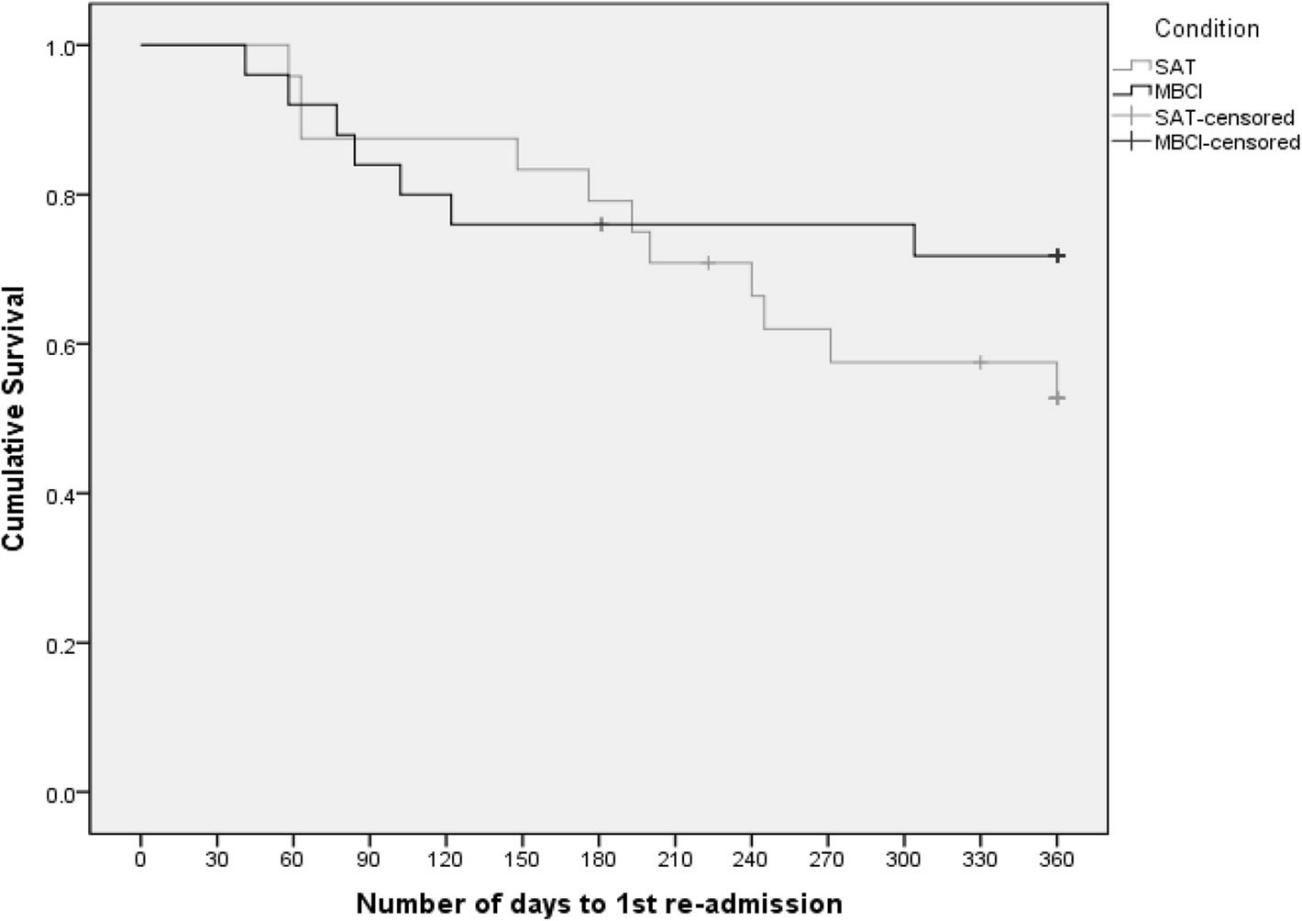
Survival curve of time to 1st admission during 12 month follow-up window: Kaplan-Meier Plot

At 12-month follow-up, there was a larger difference between re-admission rates in the MBCI and SAT groups, in favour of MBCI (MBCI = 7, SAT = 11; odds ratio = 0.46, 95% CI: 0.14–1.51). The relapse rate was slightly higher than the re-admission rate in both groups, again with the difference between groups favouring MBCI (MBCI = 9, SAT = 14; odds ratio = 0.43, 95% CI: 0.14–1.37). Figure 2 shows the Kaplan-Meier survival curve for time to readmission. This shows that the survival curves follow similar trajectories in both groups up to around 200 days post-discharge, where they begin to diverge, with the gradient of the MBCI curve flattening out, whilst the SAT curve continues downwards at a steeper rate.

## Discussion

The findings from, to our knowledge, the first RCT of a Mindfulness-Based Crisis Intervention in the UK, indicate that it is possible to recruit and retain patients with psychosis in psychological therapy trials within NHS inpatient settings, despite the challenging clinical setting. Fifty participants were recruited, which exceeded the pre-set 80% benchmark of target (*N* = 60). The follow-up rates were 98% (6 m) and 96% (12 m) for service use data extracted from clinical notes, and 86% for self-report questionnaire measures at 6 m, which also exceeded the 80% pre-set benchmark. We found that both the treatment and control therapy were highly acceptable to inpatients in terms of low drop-out rates during therapy, and high satisfaction ratings at follow-up, which is consistent with the pilot US studies of ACT for psychosis [15]. This contrasts however with findings from some of the previous UK inpatient studies offering longer courses of treatment, where treatment drop-out was high [24].

As a feasibility trial, this study was not powered to detect clinically significant differences in re-admission rates or symptom measures between the treatment and control groups. However, the outcome data are encouraging, and indicate a possible signal for a reduced risk of readmission and relapse rates in the MBCI group at 12 month follow-up. This would be consistent with the theorised mechanism of action. For example, a reduction in experiential avoidance and response style leading to a change in cognitive and behavioural responses to stressors encountered in the follow-up period post-discharge and a reduced risk of relapse/readmission. This hypothesis could be tested in a future effectiveness trial by a mediation analysis of putative mechanisms including process measures of experiential avoidance and mindfulness, which were not included in the current trial.

The findings of this study add to an increasing body of evidence that brief inpatient interventions are feasible, safe and acceptable to both patients and staff [25, 26]. Offering these brief, targeted interventions at a time of mental health crisis may be particularly helpful, as difficult thoughts and emotions are more at the surface, and people may be more open to the offer of psychological therapy [27]. This therapeutic window of opportunity may be lost as the crisis resolves, particularly for people with a ‘sealing over’ style of recovery [28], who prefer not to think about their psychotic episode after discharge.

Strengths of the current study include adherence to a pre-registered trial protocol, pre-set feasibility benchmarks for recruitment and retention, use of an active control treatment, and fidelity checking of therapist adherence and competence, in both intervention and control arms, by an independent rater. The latter is particularly important given that the same trial therapist delivered both intervention and control therapies. A recent systematic review [29] identified this as the main process giving rise to contamination in trials of complex interventions, where contamination is defined as participant in the control group receiving interventions intended only for the intervention arm [30]. Contact between people on the same ward who were assigned to different arms of the trial is not a contamination risk itself, as there is no plausible process by which a complex and individually tailored therapy could be ‘passed on’ [31].

People requiring inpatient care often have complex and chronic difficulties, and so the eligibility criteria were designed to be as broad and inclusive as possible. For example, there were no exclusions for homelessness, or co-morbid substance misuse at baseline, even though these factors can make people more challenging to follow-up post-discharge. This makes the sample more representative of the patient group the intervention would be ultimately aimed at in the future.

One of the limitations of the current study is that it was a single-therapist, single-site study, and future trials would have to establish feasibility using a multi-therapist, multi-site design. The readmission/relapse rate after discharge will also be sensitive to factors which differ across geographical areas, such as bed numbers and community care provision. Therefore the readmission rate found in this study should not be used as the sole basis for future sample size calculations, but rather a minimum clinically significant reduction in re-admission rates should be determined with key stakeholders. An additional limitation is that the authors developed the treatment manual used in the trial, which could lead to experimenter allegiance effects if the same authors evaluated the therapy in a larger clinical effectiveness trial, which could inflate the observed treatment effect [32]. However, a recent synthesis of 30 meta-analyses (240 RCTs) of allegiance effects in psychotherapy trials found that the allegiance effect was not significant for trials of interventions based on cognitive behaviour therapy, and was also not significant when treatment fidelity was properly assessed [33], as was the case for the current trial.

## Conclusions

In conclusion, this feasibility trial met all the pre-set criteria for recruitment and retention in the trial, and the interventions were acceptable to participants as shown by the lack of drop-out during the treatment phase and the high satisfaction ratings at follow-up. The study found lower readmission and relapse rates in the MBCI group at 12-month follow-up. Progression to a fully-powered clinical and cost-effectiveness trial is warranted based on these promising findings. Based on these findings, a future trial should have at least a 12 m follow-up period, as differences between the treatment and control groups did not start to emerge until this point. A future trial could also include a TAU arm, which would address the question of how MBCI compares to TAU, as well as to an active control arm (SAT) as used in this study.

## Supplementary information

**Additional file 1: Table S3.** Description of clinical outcome measures (questionnaire data). **Table S4.** Questionnaire measures (unadjusted means). **Table S5.** Coefficient estimates (B) of difference in group means at 6-month follow-up.

**Additional file 2:.** CONSORT checklist. TIDIER checklist.

## Data Availability

The datasets used and/or analysed during the current study are available from the corresponding author on reasonable request.

## Abbreviations

ACT: Acceptance and Commitment Therapy
CBTp: Cognitive-Behavioural Therapy for Psychosis
CI: Confidence Interval
CMHT: Community Mental Health Team
CRN: Clinical Research Network
HCPC: Health & Care Professions Council
ICD-10: International Classification of Diseases, version 10
ISRCTN: International Standard Randomised Controlled Trials Number
ITT: Intention to treat
KCTU: Kings Clinical Trials Unit
MBCI: Mindfulness-Based Crisis Intervention;
NHS: National Health Service
NICE: National Institute for Health and Care Excellence
OBD: Occupied bed day
RCT: Randomised Controlled Trial
SAT: Social Activity Therapy
TAU: Treatment as Usual
UK: United Kingdom
US: United States
