# Supplementary material for: Mindfulness-based crisis interventions (MBCI) for psychosis within acute inpatient psychiatric settings; a feasibility randomised controlled trial
Source: BMC Psychiatry. 2020 Apr 29;20:193. doi: 10.1186/s12888-020-02608-x (PMC7191699; doi:10.1186/s12888-020-02608-x)
Supplement: Supplementary file 1 — Additional file 1: Table S3. Description of clinical outcome measures (questionnaire data). Table S4. Questionnaire measures (unadjusted means). Table S5. Coefficient estimates (B) of difference in group means at 6-month follow-up. [file 12888_2020_2608_MOESM1_ESM.pdf]

Mindfulness-Based Crisis Interventions (MBCI) for psychosis within acute inpatient psychiatric settings; A feasibility randomised controlled trial

Supplementary material

Table 3: Description of clinical outcome measures (questionnaire data)

Construct assessed	Questionnaire	Method	Time points
Credibility of therapy	**1) Therapy credibility**	Self-report	Baseline only (immediately post-randomisation)
In the moment rating of stress and interference from symptoms, and hope for the future	**2) Stress bubbles**	Self-report	At the beginning and end of every therapy session
Frequency, distress & believability of beliefs and/or voices	**3) Self-ratings of psychotic symptoms**<br><br>(Based on Bach & Hayes, 2002; Gaudiano & Herbert, 2006)	Self-report	Baseline, end of therapy, 3 mth mid-point and 6 mth follow-up
Mood – depression, anxiety and stress	**4) DASS-21**<br><br>(Depression, anxiety & stress scale; Lovibond & Lovibond, 1995)	Self-report	Baseline, end of therapy, 3 mth mid-point and 6 mth follow-up
Self-defined recovery	**5) QPR**<br><br>(Questionnaire about the Process of Recovery; Neil et al 2009)	Self-report	Baseline, end of therapy, 3 mth mid-point and 6 mth follow-up
Voices (incl. frequency, distress, interference & compliance)	**6) HPSVQ**<br><br>(Hamilton Program for Schizophrenia Voices Questionnaire; Van Lieshout & Goldberg, 2007)	Self-report	Baseline, end of therapy, 3 mth mid-point and 6 mth follow-up
Mindfulness	**7) SMQ**<br><br>(Southampton Mindfulness Questionnaire; Chadwick et al, 2008)	Self-report	Baseline, end of therapy, 3 mth mid-point and 6 mth follow-up

1) Therapy Credibility

Immediately after randomisation, participants were read a brief description of the therapy they had been assigned to. They were then asked to rate on a scale from 0 (not helpful at all) to 10 (extremely helpful) how helpful they thought the therapy sounded.

2) Stress Bubbles

The use of within-session measures can be helpful in measuring change in brief interventions, by capturing small shifts in key processes that may occur over the course of a therapy session. Stress bubbles are a form of visual analogue scale, with 6 bubbles gradually increasing in size from “not at all” (1) to “extremely” (6). Participants rated 3 items (stress, interference from symptoms, and hope for the future) at the beginning and end of every session. These unpublished scales have been successfully used in a previous study of mindfulness interventions for psychosis (Jacobsen *et al.*, 2011).

3) Self-ratings of psychotic symptoms

This is a self-report scale that asks respondents to rate their psychotic symptoms (voices and/or distressing beliefs) on a scale of 1-7 (frequency) and 0-10 (distress and believability). These scales were used in the ACT inpatient trials (Bach and Hayes, 2002, Gaudiano and Herbert, 2006), and were found to be easy for participants to complete, and showed sensitivity to change over time.

4) Depression, anxiety and stress scales; (DASS-21) (Lovibond and Lovibond, 1995)

The DASS-21 is a short-form version of the original 42-item DASS comprising 7 items on each of the 3 sub-scales for depression, anxiety and stress. It is a self-report scale with respondents scoring each item on a four-point scale from 0 (never) to 3 (almost always). The DASS-21 has been well-validated in both clinical (Antony *et al.*, 1998) and non-clinical samples (Henry and Crawford, 2005). The DASS-21 is particularly suitable for this study, being relatively quick and easy to complete, and has been shown to have good internal consistency and convergent validity in an acute psychiatric population (Weiss *et al.*, 2015)

and is suitable for use with people experiencing psychotic symptoms (Samson and Mallindine, 2014).

5) Questionnaire about the Process of Recovery; QPR (Neil *et al.*, 2009)

The QPR is a 22-item self-report measure based on service user accounts of the process of recovery from psychosis. It has 2 sub-scales assessing both intrapersonal and interpersonal processes in recovery. Each item is rated on a 5-point scale from 0 (disagree strongly) to 4 (agree strongly). Neil et al. (2009) report that the scale has good internal consistency, construct validity and reliability.

6) Hamilton Program for Schizophrenia Voices Questionnaire; (HPSVQ) (Van Lieshout and Goldberg, 2007)

The HPSVQ is a 13-item self-report measure in which respondents rate the first 9 items on a five-point Likert scale from zero (lowest severity) to four (highest severity). The total score of these 9 items is intended to indicate the severity of auditory verbal hallucinations, and includes items on frequency, distress and interference with daily activities. There are an additional 4 qualitative items, not included for the purposes of this study. Kim et al. (Kim *et al.*, 2010) reported high test-retest reliability and good convergent validity with established clinician-rated scales (PSYRATS-AH (Haddock *et al.*, 1999b); PANSS (Kay *et al.*, 1987)) when used in a clinical sample of people with a diagnosis of schizophrenia.

7) Southampton Mindfulness Questionnaire; SMQ (Chadwick *et al.*, 2008)

The SMQ is a 16-item self-report measure designed to assess mindfulness of difficult thoughts and images. Each item is scored on a 7-point scale ranging from 0 (totally agree) to 6 (disagree totally). The SMQ has been validated in a clinical sample of people experiencing distressing psychotic symptoms. Chadwick et al. (Chadwick *et al.*, 2008) report that the SMQ has good internal reliability, and shows convergent reliability with other established mindfulness scales (e.g. MAAS; (Brown and Ryan, 2003)).

Symptom and recovery outcome measures

Tables 4 and 5 show the data from the symptom and recovery outcome measures (self-report questionnaires). In line with the analysis plan, descriptive statistics were first calculated based on unadjusted means (Table 4), before adjusting for baseline score (Table 5). Data are presented separately for beliefs (delusions) and voices for the self-rating psychotic symptom scales in both Tables 4 and 5. Participants reported delusions more commonly than voices, so the sample size is larger for the delusions ratings.

Table 4: Questionnaire measures (unadjusted means)

	T1 (Baseline)		T2 (End of therapy)		F1 (3-month follow-up)		F2 (6-month follow-up)	
	**MBCI** N=26	**SAT** N=23	**MBCI** N=23	**SAT** N=23	**MBCI** N=20	**SAT** N=20	**MBCI** N=21	**SAT** N=19
**Self-rating of psychotic symptoms (Beliefs)**								
**Frequency (1-7)**								
Mean	5.58	5.65	3.17	3.61	3.25	3.00	2.57	2.63
S.D	1.65	1.34	2.15	2.08	2.33	2.36	1.89	2.03
(95% CI)	(4.91-6.24)	(5.07-6.23)	(2.25-4.1)	(2.71-4.51)	(2.16-4.34)	(1.89-4.11)	(1.71-3.43)	(1.65-3.61)
**Distress (0-10)**								
Mean	6.69	7.70	2.83	5.26	3.70	2.90	2.24	2.16
S.D	3.42	3.42	2.82	3.40	3.94	3.43	3.21	2.97
(95% CI)	(5.31-8.07)	(6.22-9.17)	(1.61-4.05)	(3.79-6.73)	(1.86-5.54)	(1.29-4.51)	(0.78-3.70)	(0.73-3.59)
**Believability (0-10)**								
Mean	8.15	7.48	4.70	6.00	3.95	3.90	4.38	3.74
S.D	3.08	3.38	3.61	3.45	4.12	4.41	4.41	4.01
(95% CI)	(6.91-9.40)	(6.02-8.94)	(3.13-6.26)	(4.51-7.49)	(2.02-5.88)	(1.84-5.96)	(2.37-6.39)	(1.80-5.67)

	T1		T2		F1		F2	
	**MBCI** N=12	**SAT** N=12	**MBCI** N=11	**SAT** N=12	**MBCI** N=9	**SAT** N=11	**MBCI** N=9	**SAT** N=10
**Self-rating of psychotic symptoms (Voices)**								
**Frequency (1-7)**								
Mean<br>S.D<br>(95% CI)	5.08<br>1.62<br>(4.06-6.11)	5.58<br>1.5<br>(4.63-6.54)	4.09<br>2.07<br>(2.70-5.48)	3.42<br>2.11<br>(2.08-4.76)	3.00<br>2.18<br>(1.32-4.68)	2.73<br>2.15<br>(1.28-4.17)	4.22<br>2.33<br>(2.43-6.02)	2.30<br>1.95<br>(0.91-3.69)
**Distress (0-10)**								
Mean<br>S.D<br>(95% CI)	6.50<br>3.03<br>(4.57-8.43)	8.42<br>2.50<br>(6.83-10.01)	3.27<br>3.10<br>(1.19-5.36)	3.75<br>3.82<br>(1.32-6.18)	2.22<br>3.15<br>(0.00[1]-4.65)	1.18<br>2.44<br>(0.00-2.82)	3.00<br>4.03<br>(0.00-6.10)	1.80<br>2.62<br>(0.00-3.67)
**Believability (0-10)**								
Mean<br>S.D<br>(95% CI)	6.83<br>3.24<br>(4.77-8.89)	6.17<br>3.22<br>(4.12-8.21)	4.00<br>3.58<br>(1.6-6.40)	5.17<br>4.26<br>(2.46-7.87)	3.11<br>3.98<br>(0.05-6.17)	2.45<br>3.75<br>(0.00-4.97)	4.78<br>4.60<br>(1.24-8.32)	2.90<br>4.01<br>(0.03-5.77)
**HPSVQ (0-36)**<br>(Hamilton Program for Schizophrenia Voices Questionnaire)								
Mean<br>S.D<br>(95% CI)	20.67<br>8.49<br>(15.27-25.06)	25.5<br>4.78<br>(22.46-28.54)	16.45<br>8.10<br>(11.01-21.90)	13.00<br>10.87<br>(6.09-19.91)	9.44<br>8.99<br>(2.54-16.4)	8.55<br>9.13<br>(2.41-14.7)	13.63[2]<br>10.66<br>(4.71-22.5)	5.70<br>7.60<br>(0.26-11.14)

[1] Lower Bound Confidence Interval truncated to 0 where calculated value is negative to indicate floor effect

[2] N=8 in MBCI group for HPSVQ as 1 participant failed to complete all measures in the F2 assessment

	T1		T2		F1		F2	
	**MBCI**<br><br>N=24	**SAT**<br><br>N=26	**MBCI**<br><br>N=23	**SAT**<br><br>N=24	**MBCI**<br><br>N=20	**SAT**<br><br>N=21	**MBCI**<br><br>N=20[3]	**SAT**<br><br>N=20
**Mood** (DASS-21; Depression, anxiety & stress)<br><br>**Depression (0-42)**								
Mean<br>S.D<br>(95% CI)	15.77<br>12.44<br>(10.75-20.79)	20.75<br>14.62<br>(14.58-26.92)	9.04<br>10.48<br>(4.51-13.57)	15.42<br>12.28<br>(10.23-20.60)	13.7<br>10.53<br>(8.77-18.63)	11.81<br>12.18<br>(6.27-17.35)	12.60<br>11.86<br>(7.05-18.15)	13.80<br>12.66<br>(7.87-19.73)
**Anxiety (0-42)**								
Mean<br>S.D<br>(95% CI)	15.85<br>13.25<br>(10.49-21.20)	19.75<br>11.67<br>(14.82-24.68)	10.00<br>8.66<br>(6.26-13.74)	15.17<br>12.17<br>(10.03-20.31)	8.5<br>8.15<br>(4.68-12.32)	9.52<br>10.52<br>(4.73-14.31)	7.70<br>10.61<br>(2.74-12.66)	10.40<br>10.21<br>(5.62-15.18)
**Stress (0-42)**								
Mean<br>S.D<br>(95% CI)	19.31<br>11.76<br>(14.56-24.06)	25.58<br>11.57<br>(20.7-30.47)	15.04<br>9.36<br>(11.00-19.09)	17.58<br>10.23<br>(13.26-21.90)	13.50<br>11.20<br>(8.26-18.74)	9.62<br>11.57<br>(4.35-14.89)	11.40<br>11.75<br>(5.90-16.90)	12.40<br>9.37<br>(8.01-16.79)

[3] 1 MBCI participant failed to complete all questionnaire measures in the F2 assessment

	T1		T2		F1		F2	
	**MBCI**<br><br>N=24	**SAT**<br><br>N=26	**MBCI**<br><br>N=23	**SAT**<br><br>N=24	**MBCI**<br><br>N=20	**SAT**<br><br>N=21	**MBCI**<br><br>N=20	**SAT**<br><br>N=20
**Recovery (0-88)**<br><br>(QPR; Questionnaire about the Process of Recovery)<br><br>Mean<br>S.D<br>(95% CI)	65.31<br>13.25<br>(69.96-70.66)	60.58<br>18.75<br>(52.67-68.5)	67.09<br>13.94<br>(61.06-73.11)	61.00<br>13.20<br>(55.42-66.58)	63.40<br>15.04<br>(56.36-70.44)	63.52<br>15.26<br>(56.58-70.47)	62.20<br>20.45<br>(53.49-68.51)	61.00<br>16.06<br>(53.49-68.51)
	**MBCI**<br><br>N=24	**SAT**<br><br>N=26	**MBCI**<br><br>N=23	**SAT**<br><br>N=23	**MBCI**<br><br>N=20	**SAT**<br><br>N=21	**MBCI**<br><br>N=20	**SAT**<br><br>N=19[4]
**Mindfulness (0-96)**<br><br>(SMQ; Southampton Mindfulness Questionnaire)<br><br>Mean<br>S.D<br>(95% CI)	56.27<br>14.96<br>(50.23-62.31)	47.25<br>14.67<br>(41.05-53.45)	58.13<br>12.28<br>(52.82-63.44)	53.26<br>14.08<br>(47.17-59.35)	57.50<br>11.88<br>(51.94-63.06)	57.57<br>16.52<br>(50.05-65.09)	55.00<br>11.81<br>(49.47-60.53)	57.05<br>12.84<br>(50.86-63.24)

[4] 1 participant in the SAT group did not complete the SMQ at F2

Table 5: Coefficient estimates (B) of difference in group means at 6-month follow-up

	**MBCI[5] (N=21)<br>SAT (N=19)**
**Self-rating of psychotic symptoms (Beliefs)**	
**Frequency**	
**Coefficient estimate (B)<br>(95 % CI)**	-0.02<br>-1.18 to 1.15
**Distress**	
**Coefficient estimate (B)<br>(95 % CI)**	-0.46<br>-2.27 to 1.34
**Believability**	
**Coefficient estimate (B)<br>(95 % CI)**	-0.38<br>-2.69 to 1.93
	**MBCI (N=9)<br>SAT (N=10)**
**Self-rating of psychotic symptoms (Voices)**	
**Frequency**	
**Coefficient estimate (B)<br>(95 % CI)**	-2.612<br>-4.76 to -0.48
**Distress**	
**Coefficient estimate (B)<br>(95 % CI)**	-2.00<br>-5.39 to 1.38
**Believability**	
**Coefficient estimate (B)<br>(95 % CI)**	-2.33<br>-5.46 to 0.81

[5] Reference category for comparison in group means is MBCI (i.e. positive values favour MBCI)

**HPSVQ**<br><br>**(Hamilton Program for Schizophrenia Voices Questionnaire)**<br><br>**Coefficient estimate (B)**<br>**(95 % CI)**	 <br><br><br><br>-11.85<br>-20.82 to -2.89
	**MBCI (N=20)**<br>**SAT (N=20)**
**Mood**<br><br>(DASS-21; Depression, anxiety & stress)<br><br>**Depression**<br><br>**Coefficient estimate (B)**<br>**(95 % CI)**<br><br>**Anxiety**<br><br>**Coefficient estimate (B)**<br>**(95 % CI)**<br><br>**Stress**<br><br>**Coefficient estimate (B)**<br>**(95 % CI)**	 <br><br><br><br><br>1.27<br>-6.73 to 9.27<br><br><br><br>2.23<br>-4.17 to 8.63<br><br><br><br>0.71<br>-6.39 to 7.81

	**MBCI (N=20)**<br>**SAT (N=20)**
**Recovery**<br><br>(QPR; Questionnaire about the Process of Recovery)<br><br>Coefficient estimate (B)<br>(95 % CI)	 <br><br><br>1.06<br>-10.56 to 12.69
	**MBCI (N=20)**<br>**SAT (N=19)**
**Mindfulness**<br>**(0-96)**<br><br>**(SMQ; Southampton Mindfulness Questionnaire)**<br><br>**Coefficient estimate (B)**<br>**(95 % CI)**	 <br><br><br><br><br>3.92<br>-4.57 to 12.42
